# Regional ventricular enlargement as an in vivo biomarker of cumulative biomechanical neurodegeneration in former American football players

**DOI:** 10.1002/alz.71764

**Published:** 2026-08-31

**Authors:** Brian Szekely, Jared Stearns, Anya S. Mirmajlesi, Rebecca D. Folkerth, Sylvain Bouix, Daniel H. Daneshvar, Charles H. Adler, Charles Bernick, Laura J. Balcer, Jeffrey L. Cummings, Eric M. Reiman, Robert A. Stern, Martha E. Shenton, Richard J. Rushmore, Hector Arciniega, for the DIAGNOSE CTE Research Project

**Affiliations:** ^1^ Department of Rehabilitation Medicine NYU Grossman School of Medicine New York New York USA; ^2^ NYU Langone Concussion Center NYU Langone Health New York New York USA; ^3^ Brain Injury Research Center Icahn School of Medicine at Mount Sinai New York New York USA; ^4^ Department of Software Engineering and Information Technology École de technologie supérieure, Université du Québec Montréal Quebec Canada; ^5^ Department of Physical Medicine and Rehabilitation Harvard Medical School Boston Massachusetts USA; ^6^ Department of Physical Medicine and Rehabilitation Massachusetts General Hospital Boston Massachusetts USA; ^7^ Department of Physical Medicine and Rehabilitation Spaulding Rehabilitation Hospital Boston Massachusetts USA; ^8^ Department of Neurology Mayo Clinic College of Medicine, Mayo Clinic Arizona Scottsdale Arizona USA; ^9^ Cleveland Clinic Lou Ruvo Center for Brain Health Las Vegas Nevada USA; ^10^ Department of Neurology University of Washington Seattle Washington USA; ^11^ Department of Neurology NYU Grossman School of Medicine New York New York USA; ^12^ Department of Population Health NYU Grossman School of Medicine New York New York USA; ^13^ Department of Ophthalmology NYU Grossman School of Medicine New York New York USA; ^14^ Chambers‐Grundy Center for Transformative Neuroscience Department of Brain Health Kirk Kerkorian School of Medicine, University of Nevada, Las Vegas Las Vegas Nevada USA; ^15^ Banner Alzheimer's Institute and Arizona Alzheimer's Consortium Phoenix Arizona USA; ^16^ Department of Psychiatry University of Arizona Phoenix Arizona USA; ^17^ Department of Psychiatry Arizona State University Phoenix Arizona USA; ^18^ Neurogenomics Division Translational Genomics Research Institute and Alzheimer's Consortium Phoenix Arizona USA; ^19^ Department of Neurology Boston University Alzheimer's Disease Research Center and CTE Center Boston Massachusetts USA; ^20^ Department of Anatomy and Neurobiology Boston University Chobanian & Avedisian School of Medicine Boston Massachusetts USA; ^21^ Department of Neurosurgery Boston University Chobanian & Avedisian School of Medicine Boston Massachusetts USA; ^22^ Psychiatry Neuroimaging Laboratory Department of Psychiatry Brigham and Women's Hospital, Harvard Medical School Boston Massachusetts USA; ^23^ Department of Radiology Brigham and Women's Hospital, founding member of Mass General Brigham, and Harvard Medical School Boston Massachusetts USA; ^24^ Department of Psychiatry Massachusetts General Hospital, Boston, founding member of Mass General Brigham Boston Massachusetts USA; ^25^ Institute for Translational Neuroscience NYU Grossman School of Medicine New York New York USA

**Keywords:** American football, chronic traumatic encephalopathy, cumulative head impact index, neuroimaging, repetitive head impacts, structural magnetic resonance imaging, traumatic encephalopathy syndrome, ventricular volume

## Abstract

**INTRODUCTION:**

Repetitive head impacts (RHIs) have been linked to later life neurodegeneration, yet the in vivo structural correlates of cumulative biomechanical loading remain unclear. We examined whether regional ventricular morphology in former American football players reflects exposure burden and traumatic encephalopathy syndrome (TES) classification.

**METHODS:**

Participants included 170 male former football players and 54 age‐matched asymptomatic male controls from the Diagnostics, Imaging, and Genetics Network for the Objective Study and Evaluation of Chronic Traumatic Encephalopathy Research Project. Subject‐specific manual segmentation quantified lateral ventricle, inferior horn, third ventricle, and fourth ventricle volumes. Group and exposure associations were tested using generalized least squares models.

**RESULTS:**

Former players showed larger left inferior lateral ventricle volume than controls, with the largest effects among professional players. Greater cumulative linear and rotational acceleration exposure was associated with enlargement across lateral ventricular and inferior horn regions.

**DISCUSSION:**

Regional ventricular enlargement may represent an in vivo marker of cumulative biomechanical loading after RHI exposure.

## Highlights

- Former football players had larger left inferior lateral ventricle volume than controls.
- Greater cumulative acceleration exposure was associated with ventricular enlargement.
- High‐exposure players showed lateral, inferior horn, and third ventricle enlargement.

## 1 BACKGROUND

Repetitive head impacts (RHIs) sustained in contact and collision sports and military service represent a cumulative biomechanical exposure increasingly linked to later life neurodegeneration, including chronic traumatic encephalopathy (CTE).^1^, ^2^, ^3^, ^4^, ^5^, ^6^, ^7^ In American football, epidemiologic studies demonstrate a dose–response relationship between duration of play and both the likelihood of CTE and the severity of its neuropathological features at *post mortem*.^8^ Biomechanically, linear and rotational forces transmit mechanical energy to the brain, producing tissue deformation and shear strain within white matter tracts and deep brain structures.^9^, ^10^, ^11^, ^12^ Computational modeling further shows that increasing impact severity produces greater brain deformation, with rotational motion contributing substantially to strain patterns in vulnerable deep and brainstem regions.^13^, ^14^, ^15^

Clinically, individuals with substantial RHI exposure may develop progressive cognitive impairment, behavioral dysregulation, and mood disturbances later in life.^16^, ^17^, ^18^, ^19^, ^20^, ^21^, ^22^, ^23^, ^24^ Consensus criteria for traumatic encephalopathy syndrome (TES) were developed to classify progressive cognitive and neurobehavioral symptoms in the context of prior RHI exposure.^19^ However, TES is a symptom‐based research framework rather than a diagnosis of underlying neuropathology, as clinical features alone do not reliably indicate the presence or severity of CTE.^19^ This distinction underscores the need for quantitative in vivo biomarkers linking cumulative biomechanical exposure to measurable structural brain changes.

The ventricular system may represent a structural interface through which neurodegenerative processes and biomechanical forces associated with RHI converge. Ventricular enlargement is commonly interpreted as a marker of surrounding tissue loss and central brain atrophy and has been reported in CTE neuropathology.^6^, ^7^, ^20^, ^25^, ^26^, ^27^, ^28^, ^29^, ^30^, ^31^, ^32^ Ventricular anatomy may also influence the spatial distribution of mechanical forces during head impacts.^33^ Finite element modeling suggests that ventricular cavities can amplify strain in adjacent periventricular tissue during rotational loading, providing a mechanistic basis for vulnerability along ventricular boundaries.^33^ This framework is particularly relevant for midline structures such as the third ventricle, which lies adjacent to deep brain regions implicated in head injury biomechanics and trauma‐related neurodegeneration.^13^

**RESEARCH IN CONTEXT**

1. **Systematic review**: Prior studies of repetitive head impacts and chronic traumatic encephalopathy have reported ventricular enlargement and midline abnormalities in exposed athletes. However, few in vivo studies have comprehensively characterized regional ventricular morphology while evaluating both traumatic encephalopathy syndrome classification and quantitative cumulative head impact exposure.
2. **Interpretation**: This study used subject‐specific manual segmentation to evaluate lateral, inferior horn, third, and fourth ventricular volumes in former American football players and unexposed controls. Ventricular enlargement was most evident in the left inferior horn and in players with higher cumulative acceleration exposure, whereas ventricular morphology was not associated with traumatic encephalopathy syndrome classification.
3. **Future directions**: Although ventricular enlargement is not diagnostic for chronic traumatic encephalopathy, regional ventricular measures may contribute to multimodal imaging frameworks for identifying neurobiological effects of repetitive head impacts. Future longitudinal and diverse cohort studies should determine whether these markers track progression, predict clinical outcomes, and improve biomarker‐informed traumatic encephalopathy syndrome frameworks.

Evidence from experimental, in vivo, and *post mortem* studies supports ventricular and midline abnormalities after RHI exposure.^34^, ^35^ Animal models demonstrate early lateral ventricular enlargement after RHI, suggesting that ventricular expansion may represent an early structural response to cumulative biomechanical loading.^34^, ^35^ Human neuroimaging studies have reported midline abnormalities in RHI‐exposed athletes, including increased prevalence and length of the cavum septum pellucidum.^36^, ^37^, ^38^, ^39^, ^40^, ^41^, ^42^ Volumetric magnetic resonance imaging (MRI) studies in former professional football players have also shown enlargement of the lateral and inferior lateral ventricles relative to unexposed controls.^42^, ^43^, ^44^, ^45^ *Post mortem* studies further report higher frequencies of ventricular enlargement, cavum septum pellucidum, and thalamic notching among contact sport athletes with RHI exposure.^6^, ^7^, ^46^ Together, these findings suggest that ventricular and midline cerebrospinal fluid spaces may be structural correlates of cumulative RHI exposure.^22^

A key unresolved question is whether ventricular enlargement reflects cumulative biomechanical exposure rather than symptom‐based classification alone. The cumulative head impact index (CHII) estimates lifetime football‐related RHI exposure by integrating self‐reported playing history with position‐specific head impact frequencies derived from helmet sensor studies.^4^ CHII has been associated with late‐life neuropsychiatric and cognitive outcomes and may explain clinical outcomes better than simpler exposure metrics such as years of play.^16^, ^47^, ^48^ However, few in vivo studies have comprehensively characterized the ventricular system while evaluating both TES classification and quantitative exposure burden.^42^, ^49^ Examining the inferior horns of the lateral ventricles, third ventricle, and fourth ventricle may provide insight into region‐specific consequences of cumulative burden across temporal, midline, and hindbrain structures.

In the present study, we conducted a comprehensive volumetric characterization of the ventricular system in former American football players and unexposed asymptomatic controls using subject‐specific manual segmentations. We evaluated group‐level differences in ventricular morphology and examined associations with TES classification and CHII‐derived exposure burden. We hypothesized that former football players would exhibit enlargement of the lateral ventricles, inferior lateral ventricles, and third ventricle relative to controls; that ventricular volumes would be greater in TES‐positive compared to TES‐negative players; and that increasing CHII‐derived exposure would be associated with ventricular enlargement.

## 2 METHODS

### 2.1 Study design and participants

The present study used baseline data from the Diagnostics, Imaging, and Genetics Network for the Objective Study and Evaluation of Chronic Traumatic Encephalopathy (DIAGNOSE CTE) Research Project, a multi‐site observational study designed to develop in vivo biomarkers and clinical research criteria for CTE. The overall study design, recruitment strategy, inclusion and exclusion criteria, baseline assessments, and diagnostic procedures have been described previously^42^, ^50^ (see Table S1 in supporting information). Briefly, the parent cohort included former professional American football players, former college football players, and age‐matched asymptomatic unexposed male controls. For the present ventricular segmentation study, participants were excluded for missing or poor‐quality T1‐weighted/T2‐weighted MRI data or later disclosed exclusionary history. The final analytic sample included 170 former American football players, comprising 114 former professional and 56 former college players, and 54 unexposed controls. Demographic characteristics are presented in Table 1. Stratified former American football players are presented in Table S2 in supporting information. Study procedures received ethical approval from the institutional review boards (IRBs) of Boston University Medical Campus IRB #H‐34799, Mayo Clinic IRB #16‐002662, Cleveland Clinic Lou Ruvo Center for Brain Health Banner IRB #16‐1694, Alzheimer's Institute IRB #1168489, New York University Langone Health IRB# i16‐01032 CR3, and Partners Healthcare/Brigham and Women's Hospital IRB #2016P001359 and #2016P001328. A full list of the Diagnose CTE Research Project Team is presented in Table S3 in supporting information. All participants provided written informed consent before enrollment, and participants’ consent was obtained according to the Declaration of Helsinki.

**TABLE 1:** Demographics of cohort.

Characteristics	Former football players (*n* = 170)	Controls (*n* = 54)
**Age, median (IQR)**	56 (51–64)	59.5 (52.3–67)
**BMI, median (IQR) kg/m^2^**	32.2 (28.9–36.0)	30.4 (27.8–33.1)
**Years of education, median (IQR), y**	16 (16–18)	16 (16–18)
***APOE* ε4 carrier (%)**	48 (28)	10 (18.5)
**Race, n (%)**		
White	108 (63.5)	34 (63)
Black/African American	59 (34.7)	19 (35.2)
American Indian/Alaska Native	0 (0)	0 (0)
Asian	0 (0)	0 (0)
Native Hawaiian/Other Pacific Islander	0 (0)	1 (1.8)
Multiple races	3 (1.8)	0 (0)
**Exposure to RHI**		
Age of first exposure to football, mean (SD), y	11.1 (2.8)	0 (0)
Total years of football played, mean (SD), y	15.9 (4.3)	0 (0)
CHII Frequency, median (IQR) impacts x 10^3^	9.6 (7.7–13.1)	0 (0)
CHII Linear Acceleration, median (IQR) g‐force x 10^5^	2.2 (1.8–2.7)	0 (0)
CHII Rotational Force, median (IQR) radians/s^2^ x 10^7^	1.8 (1.4–2.2)	0 (0)
**Traumatic encephalopathy syndrome, *n* (%)**		
Diagnosis	108 (63.5)	0 (0)
**Levels of certainty, *n* (%)**		
No traumatic encephalopathy syndrome	61 (35.9)	0 (0)
Suggestive	32 (18.8)	0 (0)
Possible	20 (11.8)	0 (0)
Probable	56 (32.9)	0 (0)

*Notes*: Overview of demographic, genetic, and exposure characteristics for former American football players and unexposed asymptomatic controls. Values are reported as median (IQR), mean (SD), or *n* (%), as appropriate.

Abbreviations: *APOE*, apolipoprotein E; BMI, body mass index; CHII, cumulative head impact index; IQR, interquartile range; RHI, repetitive head impacts; SD, standard deviation; y, years.

### 2.2 Participant characteristics

Demographic, medical, and athletic history variables were collected as part of the previously described DIAGNOSE CTE baseline protocol.^50^ Variables used in the present analyses included age, years of education, race, body mass index (BMI), imaging site, estimated intracranial volume, and apolipoprotein E (*APOE*) ε4 carrier status (see Table 1).

### 2.3 MRI acquisition

MRI acquisition procedures followed the harmonized DIAGNOSE CTE neuroimaging protocol described previously.^42^, ^50^ Briefly, structural MRI was acquired across four imaging sites using a harmonized 3T Siemens Skyra protocol with high‐resolution 1 mm isotropic T1‐weighted magnetization‐prepared rapid gradient echo and T2‐weighted SPACE (Sampling Perfection with Application‐optimized Contrasts using different flip angle Evolution) sequences. The T1‐weighted and T2‐weighted images were used for ventricular segmentation and anatomical guidance.

### 2.4 Image processing and segmentation

Raw T1 structural images were processed with the FreeSurfer v7.1 recon‐all pipeline, which performed intensity inhomogeneity correction, intensity normalization, and skull stripping to generate brain‐extracted volumes. For each participant, the skull‐stripped T1 was rigidly reoriented to a common anterior commissure‐posterior commissure (AC‐PC)–aligned space in 3D Slicer such that the anterior and posterior commissures lay on the mid‐sagittal plane. Manual segmentations were performed in this transformed space.

Two trained raters manually segmented the left and right lateral ventricles (including the inferior horns), third ventricle, and fourth ventricle in 3D Slicer (http://www.slicer.org; version 4.10, Surgical Planning Laboratory, Brigham and Women's Hospital, Boston, MA) under the supervision of an expert neuroanatomist (R.J.R.). In this study, the inferior horns of the lateral ventricles were treated as separate regions from the rest of the lateral ventricles. Thus, the lateral ventricle measures refer to the non‐inferior horn portions of the lateral ventricles including anterior horns, bodies, atria, and occipital horns. Segmentations were guided by the Harvard–Oxford atlas based on neuroanatomical conventions for T1 MRI.^51^ In 224 brains, both raters independently traced all ventricular regions of interest. Interrater reliability was quantified with the Dice similarity coefficient between each rater and for each brain region. The Dice coefficient was the highest for the lateral ventricles (left: 0.97 ± 0.01 and right: 0.97 ± 0.02), followed by the third ventricle (0.92 ± 0.03), fourth ventricle (0.90 ± 0.03), with the lowest being the inferior horns (left: 0.72 ± 0.09 and right: 0.72 ± 0.09). All Dice coefficients exceeded the commonly used threshold for acceptable overlap (> 0.7–0.8) in manual neuroanatomical segmentation.^52^, ^53^, ^54^, ^55^ Ventricle volumes were obtained by multiplying the number of voxels by the voxel resolution size in mm^3^.

### 2.5 TES diagnosis

TES status was determined through the DIAGNOSE CTE multidisciplinary diagnostic consensus process using the National Institute of Neurological Disorders and Stroke (NINDS) Consensus Diagnostic Criteria for TES, as described previously.^19^, ^42^, ^50^ For the present analyses, former football players were classified as TES positive or TES negative based on consensus adjudication. Participants with missing TES status were excluded from TES‐specific models.

### 2.6 Exposure to RHI

Exposure to RHI was characterized using football participation history obtained from semi‐structured interviews and questionnaires. Total years of football participation were used as a measure of overall exposure burden across the playing career (from youth through college and professional play). We also evaluated the age of first exposure (or year they started playing football) to capture the potential effects of earlier participation in organized tackle football. In addition, cumulative head impact exposure was estimated using the CHII, derived from self‐reported football seasons played, position at each level of play, and published helmet accelerometer data. CHII‐based metrics reflecting estimated cumulative impact frequency, linear acceleration burden, and rotational acceleration burden. These CHII measures were examined, with higher values indicating greater estimated exposure to RHIs.^4^

### 2.7 Statistical analysis

Ventricular volume differences across exposure groups were evaluated using generalized least squares (GLS) models with covariate adjustment (see below). Group comparisons included all former football players versus unexposed controls, former professional versus former collegiate players, and each player subgroup versus controls. Lateral ventricles and inferior horns were treated as paired left–right structures. Within‐subject hemispheric correlation was modeled using a subject‐level 2 × 2 error covariance matrix estimated from hemisphere‐specific residuals, with fixed effects for group, hemisphere, and group‐by‐hemisphere interaction. Third and fourth ventricular volumes were analyzed using univariate GLS models. All models were adjusted for age, BMI, race, years of education, imaging site, estimated total intracranial volume (eTIV), and *APOE* ε4 status. Statistical uncertainty was quantified using subject‐level bootstrap resampling (1000 iterations), with percentile bootstrap confidence intervals and *p* values derived under a normal approximation. Multiple comparisons across ventricular outcomes were controlled using the Benjamini–Hochberg false discovery rate (FDR) procedure (*p* < 0.05).

Among former football players, we examined associations between ventricular volumes and TES classification (TES positive vs. TES negative). Participants with missing TES status were excluded (*n* = 1). Separate logistic regression models were fitted for each ventricular region, with TES status as the outcome and ventricular volume as the predictor. Models were adjusted for age, BMI, years of education, race, imaging site, eTIV, *APOE* ε4 carrier status, and level of play (collegiate vs. professional). To account for multiple regional tests, *p* values were controlled using the Benjamini–Hochberg FDR.

Among former football players, associations between cumulative head impact exposure and ventricular volumes were evaluated using the same GLS framework described above. Exposure predictors were examined separately and included CHII‐derived impact frequency, linear acceleration burden, rotational acceleration burden, and age of first exposure. An exploratory analysis was also performed in which the former football cohort was stratified into low‐ and high‐exposure groups using the 50th percentile of the summed CHII linear and rotational acceleration burden data.

## 3 RESULTS

### 3.1 Demographic factors

The Welch two‐sample t test showed a significant difference in BMI between former American football players and unexposed asymptomatic controls, with the football group having a higher BMI (*t*[90.3] = 2.3, mean difference = 1.7, 95% confidence interval [CI]: 0.2 to 3.1, P = 0.024). No other demographic variables differed significantly between groups (see Table 1).

### 3.2 Ventricular volume differences between former football players and asymptomatic controls

**FIGURE 1:**
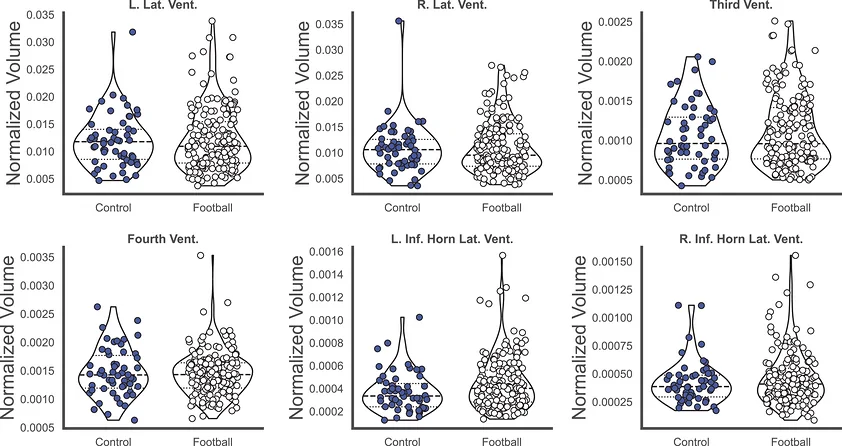
Ventricular volumetric differences in controls compared to former American football players. Group‐level comparisons between the unexposed control group and former American football players in the left/right lateral ventricle, third ventricle, fourth ventricle, and left/right inferior horn of the lateral ventricle. A significant group difference was observed in the left inferior horn of the lateral ventricle, but not in any other regions. A blue circle indicates controls and the clear circle indicate the football cohort. Inf., inferior; L, left; Lat, lateral; R, right; Vent., ventricle.

**FIGURE 2:**
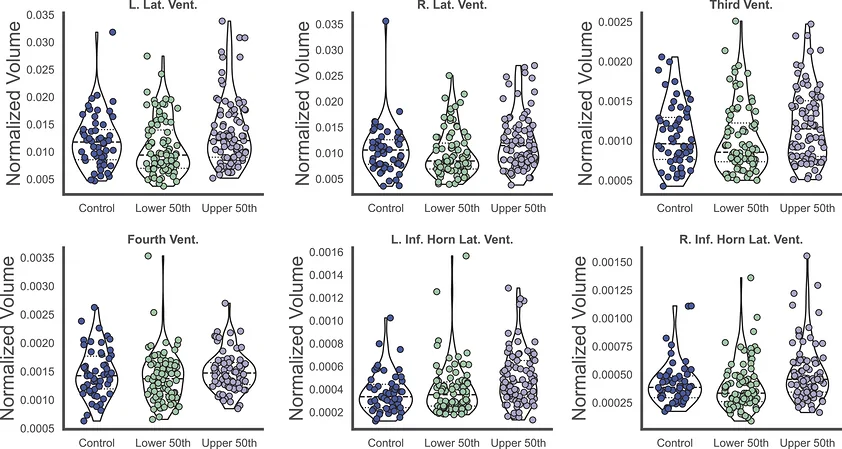
Summed cumulative exposure metrics for percentile groups. Group‐level comparisons between the unexposed control group, lower and upper 50th former American players percentile groups in the left/right lateral ventricle, third ventricle, fourth ventricle, and left/right inferior horn of the lateral ventricle. A significant group difference was observed in the bilateral lateral ventricles, bilateral inferior horns of the lateral ventricles and third ventricle, but not in the fourth ventricle. A blue circle indicates the controls, light green indicates former players below the 50th percentile group, and light purple indicates former players above the 50th percentile group. Inf., inferior; L, left; Lat, lateral; R, right; Vent., ventricle.

Using the GLS model, we found that former football players had significantly larger left inferior horn of the left lateral ventricle volume than asymptomatic controls (95% CI: −199.66, −47.70; *p* = 0.004), whereas no other regions survived FDR correction (all *p* > 0.08). In post hoc subgroup analyses, professional and collegiate players did not differ in any region after FDR correction (all *p* > 0.19), although the third ventricle differed at the uncorrected level (95% CI: −144.09, −6.42; *p* = 0.032). Collegiate players also did not differ from controls after FDR correction (all *p* > 0.19), with only an uncorrected increase in third ventricle volume (95% CI: −147.91, −6.66; *p* = 0.032). In contrast, professional football players had significantly greater left inferior horn volume of the left lateral ventricle than controls (95% CI: −220.44, −55.03; *p* = 0.005) after FDR correction. The right inferior horn (95% CI: −187.71, −4.74; *p* = 0.041) and third ventricle (95% CI: −147.44, −5.92; *p* = 0.035) were significant only before FDR correction. All other regions were not significant after correction. In an exploratory post hoc stratified analysis, former football players were divided at the 50th percentile of the summed cumulative linear and rotational acceleration exposure metrics. Relative to controls, the subgroup above the median exposure showed higher lateral ventricle, inferior horn, and third ventricle volumes, including the left lateral ventricle (95% CI: –3827.10, 11.70; P < 0.032), right lateral ventricle (95% CI: –3830.30, 117; P < 0.032), left inferior horn (95% CI: −274.97, −110.84; *p* < 0.001), right inferior horn (95% CI: −256.12, −59.59; *p* = 0.002), and third ventricle (95% CI: −425.33, −123.45; *p* < 0.001). The fourth ventricle did not differ from controls (95% CI: −203.21, 128.13; *p* = 0.315). In contrast, the subgroup below the median exposure did not differ from controls in any ventricular region after FDR correction (all *p* ≥ 0.826). See Figure 1, Figure 2, Table 2, and Table 3 for detailed distributions across ventricles.

**TABLE 2:** Ventricular volume comparisons.

Condition and region of interest	Estimate	95% CI	*p* uncorrected	*p* corrected
**Former players vs. unexposed asymptomatic controls**				
Left lateral ventricle	−475.35	[2081.35, 1196.00]	0.58	0.58
Right lateral ventricle	−854.77	[−2494.05, 983.41]	0.34	0.44
Left inf. horn lat. vent.	−125.00	[−199.66, –47.70]	0.0008	0.004
Right inf. horn lat. vent.	−80.22	[−162.01, 7.72]	0.059	0.098
Third ventricle	−146.78	[−275.83, –13.60]	0.032	0.08
Fourth ventricle	−15.01	[−172.50, 145.28]	0.85	0.71
**Former professional players vs. unexposed asymptomatic controls**				
Left lateral ventricle	−437.17	[−2234.70, 1390.29]	0.64	0.77
Right lateral ventricle	−673.27	[−2488.95, 1307.70]	0.49	0.73
Left inf. horn lat. vent.	−139.05	[−220.44, –55.03]	0.0001	0.005
Right inf. horn lat. vent.	−97.48	[−187.71, –4.74]	0.041	0.08
Third ventricle	−76.22	[−147.44, –5.92]	0.035	0.08
Fourth ventricle	−9.94	[−82.70, 65.89]	0.79	0.79
**Former collegiate players vs. former professional players**				
Left lateral ventricle	−540.76	[−2488.52, 1481.92]	0.59	0.88
Right lateral ventricle	−130.55	[−1802.35, 1525.66]	0.88	0.88
Left inf. horn lat. vent.	−20.78	[−99.30, 59.11]	0.60	0.88
Right inf. horn lat. vent.	−25.71	[−107.04, 53.08]	0.53	0.88
Third ventricle	−76.60	[−144.10, –6.42]	0.032	0.19
Fourth ventricle	−10.28	[−84.60, 63.23]	0.79	0.88
**Former collegiate players vs. unexposed asymptomatic controls**				
Left lateral ventricle	62.10	[−1938.86, 2063.16]	0.95	0.95
Right lateral ventricle	586.69	[−2519.71, 1405.86]	0.56	0.90
Left inf. horn lat. vent.	−68.58	[−146.17, 14.46]	0.10	0.30
Right inf. horn lat. vent.	−21.11	[−96.73, 61.34]	0.60	0.90
Third ventricle	−77.10	[−147.91, –6.66]	0.032	0.19
Fourth ventricle	−10.16	[−81.33, 62.93]	0.79	0.94

*Notes*: Group‐level comparisons of ventricular volumes between former American football players and unexposed asymptomatic controls, including subgroup comparisons by level of play. Estimates, 95% confidence intervals, uncorrected *p* values, and multiple‐comparison corrected *p* values are shown for the bilateral lateral ventricles, bilateral inferior horns of the lateral ventricles, third ventricle, and fourth ventricle.

Abbreviations: CI, confidence interval; Inf, inferior; Lat, lateral; Vent, ventricle.

**TABLE 3:** CHII burden percentiles.

Condition and Region of Interest	Estimate	95% CI	*p* uncorrected	*p* corrected
**>50th former players vs. unexposed asymptomatic controls**				
Left lateral ventricle	−1949	[−3827.1, 11.7]	0.044	0.032
Right lateral ventricle	−1939	[−3830.3, 117]	0.053	0.032
Left inf. horn lat. vent.	−191	[−275, –111]	0.000008	0.000024
Right inf. horn lat. vent.	−158.3	[−256.1, –60]	0.0019	0.0019
Third ventricle	−272.1	[−425.3, –123.4]	0.000395	0.000592
Fourth ventricle	−40.3	[−203, 128.1]	0.63	0.32
**<50th former players vs. unexposed asymptomatic controls**				
Left lateral ventricle	1046	[−782, 2919]	0.28	0.83
Right lateral ventricle	280.4	[−1505, 2348]	0.78	0.99
Left inf. horn lat. vent.	−48.4	[−132.1, 39.4]	0.26	0.83
Right inf. horn lat. vent.	5.0	[−72, 89.2]	0.90	0.99
Third ventricle	−25.9	[−179, 116]	0.73	0.99
Fourth ventricle	0.61	[−198, 197.2]	0.99	0.99

Notes: Ventricular volume comparisons between unexposed asymptomatic controls and former American football players stratified by the 50th percentile of summed cumulative linear and rotational acceleration exposure. Estimates, 95% confidence intervals, uncorrected *p* values, and corrected *p* values are shown for each ventricular region.

Abbreviations: CHII, cumulative head impact index; CI, confidence interval; Inf, inferior; Lat, lateral; Vent, ventricle.

### 3.3 Ventricular volumes and TES status

Using logistic regression, we evaluated whether ventricular volumes were associated with TES status (TES positive vs. TES negative) among our cohort of former American football players (*n *= 170) and found that no ventricular volume was significantly associated with TES status (*p* > 0.17; Table 4).

**TABLE 4:** Exposure metrics and traumatic encephalopathy syndrome.

Exposure and TES	Estimate	95% CI	*p* uncorrected	*p* corrected	R squared
**Age of first exposure**				
Left lateral ventricle	−255	[600, 124]	0.18	0.22	—
Right lateral ventricle	−251	[−548, 52]	0.12	0.22	—
Left inf. horn lat. vent.	−15.2	[−27.8, –1.4]	0.021	0.12	—
Right inf. horn lat. vent.	−10.6	[−25.5, 4.1]	0.16	0.22	—
Third ventricle	−24.3	[−50.3, 1.2]	0.07	0.21	—
Fourth ventricle	−13.0	[−43.7, 15.2]	0.39	0.39	—
**CHII frequency**					
Left lateral ventricle	0.19	[–0.01, 0.42]	0.092	0.14	
Right lateral ventricle	0.17	[0.0004, 0.37]	0.085	0.14	
Left inf. horn lat. vent.	0.008	[–0.0008, 0.017]	0.083	0.14	
Right inf. horn lat. vent.	0.008	[–0.0002, 0.017]	0.060	0.14	
Third ventricle	0.007	[−0.008, 0.023]	0.36	0.43	
Fourth ventricle	0.002	[−0.012, 0.018]	0.76	0.76	—
**CHII linear acceleration**					
Left lateral ventricle	0.021	[0.009, 0.035]	0.001	0.003	—
Right lateral ventricle	0.017	[0.006, 0.029]	0.003	0.004	—
Left inf. horn lat. vent.	0.001	[0.0005, 0.0017]	0.0003	0.0009	—
Right inf. horn lat. vent.	0.001	[0.0006, 0.0018]	0.00007	0.0004	—
Third ventricle	0.001	[0.0001, 0.002]	0.024	0.029	—
Fourth ventricle	0.0007	[−0.0004, 0.0017]	0.21	0.21	—
**CHII rotational force**					
Left lateral ventricle	0.0002	[0.00006, 0.0004]	0.011	0.022	—
Right lateral ventricle	0.0002	[0.00003, 0.0003]	0.019	0.028	—
Left inf. horn lat. vent.	0.00001	[0.000004, 0.00002]	0.001	0.004	—
Right inf. horn lat. vent.	0.00001	[0.000006, 0.00002]	0.0002	0.001	—
Third ventricle	0.00001	[0.000001, 0.00003]	0.047	0.056	—
Fourth ventricle	0.000006	[–0.000008, 0.00002]	0.36	0.36	—
**TES**					—
Left lateral ventricle	0.00008	[0.000007, 0.0002]	0.031	0.17	0.16
Right lateral ventricle	0.00005	[0.00003, 0.0001]	0.20	0.40	0.14
Left inf. horn lat. vent.	0.002	[−0.00005, 0.004]	0.06	0.17	0.15
Right inf. horn lat. vent.	0.0007	[−0.0007, 0.002]	0.30	0.45	0.14
Third ventricle	0.00004	[−0.0008, 0.0009]	0.92	0.92	0.13
Fourth ventricle	0.0004	[−0.0005, 0.001]	0.38	0.45	0.14

Notes: Associations between ventricular volumes and age of first exposure, CHII frequency, CHII linear acceleration, CHII rotational force, and traumatic encephalopathy syndrome status among former American football players. Estimates, 95% confidence intervals, uncorrected *p* values, corrected *p* values, and model R‐squared values are reported where applicable.

Abbreviations: CHII, cumulative head impact index; CI, confidence interval; Inf, inferior; Lat, lateral; TES, traumatic encephalopathy syndrome; Vent, ventricle.

### 3.4 Cumulative head impact burden and first exposure

Using the GLS framework previously used, we assessed how ventricular volumes were associated with CHII metrics and the year started playing football (age of first exposure) within former American football players. For CHII‐Frequency, no associations survived FDR correction (*p* > 0.14). There were trend‐level positive associations for the lateral ventricles and inferior horns of the lateral ventricles, but these did not remain significant after correction. For CHII Linear Acceleration, higher exposure was associated with larger ventricular volumes in multiple regions. Specifically, greater CHII Linear Acceleration was associated with larger left lateral ventricle volume (95% CI: 0.009, 0.035; *p* = 0.003), right lateral ventricle volume (95% CI: 0.006, 0.029; *p* = 0.004), left inferior horn of the lateral ventricle volume (95% CI: 0.0005, 0.0017; *p* < 0.001), right inferior horn of the lateral ventricle volume (95% CI: 0.0006, 0.0018; *p* < 0.001), and third ventricle volume (95% CI: 0.0001, 0.002; *p* = 0.029). The fourth ventricle had no significant association with CHII Linear Acceleration (*p* = 0.21). For CHII Rotational Force, higher exposure was also associated with larger ventricular volumes, primarily in the lateral ventricles and the inferior horn of the lateral ventricles. Greater CHII Rotational Force was associated with larger left lateral ventricle volume (95% CI: 0.00006, 0.0004; *p* = 0.022), right lateral ventricle volume (95% CI: 0.00003, 0.0003; *p* = 0.028), left inferior horn of the lateral ventricle volume (95% CI: 0.000004, 0.00002; *p* = 0.004), and right inferior horn of the lateral ventricle (95% CI: 0.000006, 0.00002; *p* = 0.001). The association with third ventricle and fourth ventricle volume was not significant after FDR correction (*p* = 0.056, *p* = 0.36). For the age of first exposure, no associations with volume across any region survived FDR correction (all *p* > 0.12). See Table 4 for detailed results.

## 4 DISCUSSION

In this study, we evaluated ventricular morphology in former American football players with varying levels of RHI exposure and compared them to unexposed asymptomatic controls. Former football players exhibited significantly larger left inferior lateral ventricle volume relative to controls, with the largest effects observed in former professional athletes. In addition, cumulative head impact burden was associated with ventricular enlargement across multiple ventricular compartments. Greater linear acceleration exposure was associated with broader ventricular enlargement, while rotational exposure was associated with enlargement of the lateral ventricles and their inferior horns. Exploratory stratification by combined linear and rotational acceleration burden further supported a dose‐dependent relationship between cumulative biomechanical loading and ventricular enlargement. Importantly, ventricular morphology was not associated with TES classification, suggesting that ventricular enlargement may more closely reflect cumulative biomechanical exposure than symptom‐based clinical categorization. Although *APOE* ε4 status is relevant to neurodegeneration risk in RHI populations, *APOE* ε4 was not significantly associated with ventricular volume in the GLS models, suggesting that the ventricular findings were not primarily driven by *APOE* ε4 status.

One of the most notable findings was enlargement of the inferior lateral ventricles in former football players, with the largest effects observed among professional athletes. Both inferior horns showed a similar direction in that the inferior horns were larger in the former football players compared to the controls, suggesting that the findings are more consistent with regional enlargement of the inferior horns than with a preferential effect on the left cerebral hemisphere. Due to many prior studies analyzing lateral ventricular volume as a bilateral or total measure, the asymmetric regional findings may be obscured.^56^ The inferior horns lie adjacent to medial temporal lobe structures, including the hippocampus and amygdala, regions that are particularly vulnerable to neurodegenerative processes.^2^, ^3^ In this cohort of former American football players, we have previously reported volumetric reductions in both the hippocampus and amygdala, suggesting that enlargement of the inferior horns may reflect structural loss within adjacent medial temporal structures.^42^, ^45^ Ventricular expansion in this context is commonly interpreted as a secondary marker of surrounding tissue loss and central atrophy.^44^ However, ventricular enlargement should not be interpreted as specific neurodegeneration or atrophy alone. Axonal injury and periventricular white matter alterations may also contribute to ventricular expansion after RHI exposure. Recent diffusion findings in this cohort demonstrate that there is higher fractional anisotropy in former football players relative to controls.^57^ Furthermore, within football players, greater cumulative RHI exposure was associated with lower fractional anisotropy, suggesting that exposure‐related white matter alterations may contribute to ventricular morphology without indicating widespread white matter abnormality at the group level.^59^ The finding that ventricular differences were primarily driven by former professional players rather than collegiate players suggests that cumulative exposure associated with higher levels of play may contribute to greater structural brain changes. This interpretation is consistent with prior in vivo and *post mortem* findings demonstrating that longer duration of football exposure and higher levels of play are associated with increased odds of CTE and greater neuropathologic severity.^22^, ^58^ Together, these observations suggest that RHI‐related neurodegeneration may manifest most prominently as focal atrophy in medial temporal structures bordering the inferior horns, including the hippocampus, amygdala, and parahippocampal regions, rather than as uniform expansion of the ventricular system.^2^, ^42^ In this context, global volumetric measures of the lateral ventricles may obscure spatially localized changes in ventricular morphology.^25^ Ventricular shape analyses may therefore provide a more sensitive approach for detecting regional deformation patterns that reflect focal neurodegeneration in adjacent anatomical structures.^25^ Overall, this interpretation is consistent with our prior structural MRI work demonstrating volumetric reductions in medial temporal lobe structures.^42^, ^45^

At the group level, we also observed enlargement of the third ventricle in former football players relative to controls, with a consistent pattern across exposure groups, although this effect did not survive correction for multiple comparisons. The third ventricle is a midline cerebrospinal fluid cavity bordered by deep gray matter nuclei, including the thalamus and hypothalamus, regions implicated in the neuropathology of CTE, in which neuronal loss and gliosis have been reported in surrounding structures.^2^, ^3^ Alterations in deep midline structures are commonly described in CTE and other trauma‐related neurodegenerative processes, whereas neurodegeneration in Alzheimer's disease more prominently affects medial temporal and cortical regions.^2^, ^3^, ^44^ Enlargement of the third ventricle may therefore reflect structural changes within adjacent deep‐brain nuclei associated with RHI exposure.^2^ Considered alongside enlargement of the inferior lateral ventricles adjacent to medial temporal structures, these findings suggest that ventricular alterations in former football players may reflect focal neurodegeneration affecting both medial temporal and deep midline compartments, rather than uniform ventricular expansion.^2^, ^25^, ^42^, ^44^

This interpretation was strengthened by the exploratory post hoc stratified analysis based on cumulative RHI burden. When former football players were divided at the median of the summed linear and rotational acceleration exposure metrics, only the higher exposure subgroup demonstrated significant enlargement of the lateral ventricles, inferior horns, and third ventricle relative to controls. This threshold‐like pattern suggests that ventricular expansion may emerge primarily in the setting of greater cumulative biomechanical loading, supporting a dose‐dependent relationship between RHI exposure and structural alterations of cerebrospinal fluid spaces. The presence of enlargement across both lateral ventricular compartments and the third ventricle in the higher exposure subgroup also reinforces the possibility that cumulative exposure preferentially affects medial temporal and deep periventricular regions, where tissue loss may be reflected as secondary ventricular expansion. These findings suggest that gross ventricular enlargement may emerge only after cumulative biomechanical exposure exceeds a critical threshold, paralleling the dose‐dependent increase in CTE burden and neuropathological severity observed with greater RHI exposure in *post mortem* cases.^4^, ^5^, ^6^, ^8^, ^59^

Among former American football players, we did not observe significant differences in ventricular volumes between TES‐positive and TES‐negative individuals after correction for multiple comparisons. This finding suggests that ventricular morphology may not be strongly associated with symptom‐based TES classification within this cohort. TES was developed as a clinical research framework to identify individuals with cognitive impairment, behavioral dysregulation, or mood symptoms in the context of prior RHI exposure.^19^ However, TES criteria rely primarily on clinical symptoms and functional decline rather than direct markers of underlying neuropathology.^19^ As a result, individuals meeting TES criteria may represent a heterogeneous group with varying underlying biological substrates, including neurodegenerative processes related to RHI exposure as well as other age‐related conditions.^3^, ^19^ The absence of ventricular differences between TES‐positive and TES‐negative players highlights a broader challenge in the field: symptom‐based clinical frameworks may not reliably capture the underlying structural brain alterations associated with cumulative RHI exposure.^3^, ^19^, ^22^ These findings suggest that the relationship between clinical presentation and structural brain changes in RHI‐exposed populations is complex and may not map directly onto symptom‐based frameworks such as TES.^3^, ^19^ Objective measures such as neuroimaging, fluid biomarkers, and exposure metrics may therefore provide complementary information beyond clinical classification alone.^4^, ^47^, ^60^

Ventricular morphology was also associated with cumulative head impact exposure. Greater estimated CHII linear acceleration exposure was associated with larger volumes of the bilateral lateral ventricles, inferior horns, and third ventricle, whereas greater rotational acceleration exposure was associated with enlargement of the bilateral lateral ventricles and inferior horns. These findings suggest that ventricular expansion may reflect cumulative biomechanical strain affecting adjacent brain regions and that acceleration‐based exposure metrics capture neurobiologically relevant aspects of RHI burden more effectively than impact counts alone.^4^, ^33^ These observations are consistent with prior DIAGNOSE CTE findings, in which CHII linear and rotational head impact exposures were associated with increased perivascular space volume, whereas impact frequency was not.^42^, ^45^, ^60^, ^61^ Similarly, CHII rotational acceleration has been linked to greater cavum septum pellucidum length, supporting the sensitivity of cerebrospinal fluid–related markers to cumulative RHI exposure.^49^

Several limitations should be considered. First, the cohort included only men with American football exposure, limiting generalizability to women, other contact‐sport athletes, and newer football eras. Second, the asymptomatic control group and demographic subgroup differences may have influenced comparisons despite covariate adjustment. Third, CHII was estimated from self‐reported football history, position, and published helmet‐sensor data rather than direct player‐level recordings, potentially introducing exposure misclassification. Some CTE‐relevant brain regions cannot be reliably quantified in vivo with standard structural MRI methods, limiting anatomical coverage. Finally, because scans were acquired years after football ended and *post mortem* confirmation was unavailable, these findings cannot be temporally linked to RHI exposure, tied directly to CTE pathology, or separated from other neurodegenerative or cerebrovascular processes.

## 5 CONCLUSION

In this study, former American football players exhibited larger ventricular volumes compared to unexposed asymptomatic controls, with ventricular enlargement associated with cumulative head impact exposure metrics. These findings suggest that RHI exposure may contribute to structural brain changes reflected by expansion of ventricular cerebrospinal fluid spaces, consistent with neurodegenerative processes affecting adjacent brain regions. Importantly, ventricular morphology did not differ between TES‐positive and TES‐negative players, indicating that symptom‐based TES classification may not fully capture structural brain alterations associated with RHI exposure. Together, these findings support the potential utility of quantitative neuroimaging markers for detecting in vivo brain changes related to cumulative head impact exposure. Future research should evaluate whether integrating validated imaging biomarkers into TES frameworks improves the identification of individuals with greater neurobiological effects of RHI.

## CONFLICT OF INTEREST STATEMENT

Competing interest are disclosed in ICMJE disclosure of interest forms. Author disclosures are available in the Supporting Information.

## CONSENT STATEMENT

The study was approved by the institutional review boards at all participating sites, including Boston University Medical Campus, Mayo Clinic, Cleveland Clinic Lou Ruvo Center for Brain Health, Banner Alzheimer's Institute, NYU Langone Health, and Partners Healthcare/Brigham and Women's Hospital. All participants provided written informed consent prior to enrollment, and all study procedures were conducted in accordance with the Declaration of Helsinki.

## Supporting information



Supporting Information

Supporting Information
