# Supplementary material for: Regional ventricular enlargement as an in vivo biomarker of cumulative biomechanical neurodegeneration in former American football players
Source: Alzheimers Dement. 2026 Aug 31;22(9):e71764. doi: 10.1002/alz.71764 (PMC13529812; doi:10.1002/alz.71764)
Supplement: Supplementary file 2 [file ALZ-22-e71764-s002.docx]

Supplemental Information

Supplemental Table 1 Inclusion and Exclusion Criteria of Diagnose CTE Research Project Cohort

| Participants | Inclusion Criteria | Exclusion Criteria |
| --- | --- | --- |
| Former College Football player group (COL) [N=60] | | |
|  | Played for more than or equal to 6 years of organized American football including more than or equal to 3 years of college varsity level play | Primary athletic position was quarterback, kicker |
|  | Played at least one of the following positions in college: offensive lineman, defensive lineman, linebacker, offensive back or receiver, or defensive back |  |
|  | No organized American football or contact/collision sports following college |  |
| Former professional football player group (PRO) [N=120] | | |
|  | Played for more than or equal 12 years of organized football this includes (more than equal to 3 years in college and more than 3 years in the NFL) | Primary athletic position was quarterback, kicker |
|  | Played at least one of the following positions: offensive lineman, defensive lineman, linebacker, offensive back or receiver, or defensive back |  |
| Unexposed Asymptomatic Controls [N=60] | | |
|  | No history of participation in organized contact/collision sports or military combat service or training | History of TBI or concussion |
|  | Asymptomatic, based on telephone screening questions (asked of both participant and informant) regarding current mood, behavior, or cognitive symptoms, as well as functional dependence | History of formal diagnosis or treatment of psychiatric illness or cognitive impairment |
|  | Have at least 2 years of post-secondary education at a 4-year accredited college/university, or have an associate’s degree | Body mass index (BMI) < 24 |

COL = College; PRO = Professional; BMI = Body Mass Index

Supplemental Table 2 Stratified Cohort Demographics

| Stratified Cohort Demographics | Former professional players (n=114) | Former college players (n=56) | |
| --- | --- | --- | --- |
| Characteristics | | |  |
| Age, median [IQR] | 60 (53-65) | 51 (48-56.5) |  |
| BMI, median [IQR], kg/m^2^ | 31.2 (28.8-34.3) | 33.2 (29.2-37.5) |  |
| Years of education, median [IQR], y | 16 (16-17) | 16 (16-18) |  |
| APOE ε4 carrier (%) | 30 (26) | 18 (32) |  |
| Race, n (%) |  |  |  |
| White | 64 (56) | 44 (79) |  |
| Black/African American | 48 (42) | 11 (20) |  |
| American Indian/Alaska Native | 0 (0) | 0 (0) |  |
| Asian | 0 (0) | 0 (0) |  |
| Native Hawaiian/Other Pacific Islander | 0 (0) | 0 (0) |  |
| Multiple Races | 2 (2) | 1 (1) |  |
| Exposure to RHI |  |  |  |
| Age of first exposure {SD}, y | 11.6 (2.8) | 10.1 (2.7) |  |
| CHII Frequency, median [IQR] impacts x 10^3^ | 11.0 (8.5-15.3) | 8.3 (6.5-10.1) |  |
| CHII Linear Acceleration, median [IQR] g-force x 10^5^ | 2.3 (2.0-2.9) | 1.8 (1.5-2.3) |  |
| CHII Rotational Force, median [IQR] radians^2^ x 10^7^ | 2.0 (1.6-2.4) | 1.4 (1.0-1.7) |  |

Values are displayed as median unless otherwise specified. IQR = inter quartile range; APOE = Apolipoprotein ε4 allele; SD = Standard deviation; y = Years

Supplemental Table 3 Diagnose CTE Research Project Team

| Name | | Academic Degrees | Institution | Location | Role or Contribution | |
| --- | --- | --- | --- | --- | --- | --- |
| Yi Su | Ph.D. | | Banner Alzheimer’s Institute | Phoenix, Arizona, US | Contributor |  |
| Kewei Chen | Ph.D. | | Banner Alzheimer’s Institute | Phoenix, Arizona, US | Contributor |  |
| Hillary Protas | Ph.D. | | Banner Alzheimer’s Institute | Phoenix, Arizona, US | Contributor |  |
| Conni Boker | M.B.A. | | Banner Alzheimer’s Institute | Phoenix, Arizona, US | Contributor |  |
| Rhoda Au | Ph.D. | | Boston University School of Medicine | Boston, Massachusetts, US | Contributor |  |
| Robert C. Cantu | Ph.D. | | Boston University School of Medicine | Boston, Massachusetts, US | Contributor |  |
| Lindsay Farrer | Ph.D. | | Boston University School of Medicine | Boston, Massachusetts, US | Contributor |  |
| Robert Helm | M.D. | | Boston University School of Medicine | Boston, Massachusetts, US | Contributor |  |
| Neil Kowall | M.D. | | Boston University School of Medicine | Boston, Massachusetts, US | Contributor |  |
| Gustavo Mercier | M.D., Ph.D. | | Boston University School of Medicine | Boston, Massachusetts, US | Contributor |  |
| James Otis | M.D. | | Boston University School of Medicine | Boston, Massachusetts, US | Contributor |  |
| Jason Weller | M.D. | | Boston University School of Medicine | Boston, Massachusetts, US | Contributor |  |
| Irene Simkin | M.S. | | Boston University School of Medicine | Boston, Massachusetts, US | Contributor |  |
| Alondra Andino | B.A. | | Boston University Project Coordinating Center Staff | Boston, Massachusetts, US | Coordination |  |
| Shannon Conneely | B.A. | | Boston University Project Coordinating Center Staff | Boston, Massachusetts, US | Coordination |  |
| Courtney Diamond | M.B.A. | | Boston University Project Coordinating Center Staff | Boston, Massachusetts, US |  |  |
| Olivia Haller | B.A. | | Boston University Project Coordinating Center Staff | Boston, Massachusetts, US | Coordination |  |
| Tennyson Hung | M.B.A. | | Boston University Project Coordinating Center Staff | Boston, Massachusetts, US | Coordination |  |
| Nicole Gullotti | M.B.A. | | Boston University Project Coordinating Center Staff | Boston, Massachusetts, US | Coordination |  |
| Megan Mariani | B.S., B.A. | | Boston University Project Coordinating Center Staff | Boston, Massachusetts, US | Coordination |  |
| Brian Mayville | B.S., B.A. | | Boston University Project Coordinating Center Staff | Boston, Massachusetts, US | Coordination |  |
| Kathleen McLaughlin | B.A. | | Boston University Project Coordinating Center Staff | Boston, Massachusetts, US | Coordination |  |
| Mary Nanna | B.A. | | Boston University Project Coordinating Center Staff | Boston, Massachusetts, US | Coordination |  |
| Taylor Platt | M.P.H. | | Boston University Project Coordinating Center Staff | Boston, Massachusetts, US | Coordination |  |
| Sury Pulukuri | B.A. | | Boston University Project Coordinating Center Staff | Boston, Massachusetts, US | Coordination |  |
| Fiona Rica | M.P.H. | | Boston University Project Coordinating Center Staff | Boston, Massachusetts, US | Coordination |  |
| Madison Sestak | B.S. | | Boston University Project Coordinating Center Staff | Boston, Massachusetts, US | Coordination |  |
| Michael McClean | Sc.D. | | Boston University School of Public Health | Boston, Massachusetts, US | Contributor |  |
| Douglas Annis | M.S. | | Boston University School of Public Health | Boston, Massachusetts, US | Contributor |  |
| Christine Chaisson | M.P.H. | | Boston University School of Public Health | Boston, Massachusetts, US | Contributor |  |
| Carolyn Finney | B.A. | | Boston University School of Public Health | Boston, Massachusetts, US | Contributor |  |
| Kerrin Gallagher | M.P.H | | Boston University School of Public Health | Boston, Massachusetts, US | Contributor |  |
| Kaitlin Hartlage | M.P.H | | Boston University School of Public Health | Boston, Massachusetts, US | Contributor |  |
| Jun Lu | M.S. | | Boston University School of Public Health | Boston, Massachusetts, US | Contributor |  |
| Brett Martin | M.S. | | Boston University School of Public Health | Boston, Massachusetts, US | Contributor |  |
| Emmanuel Ojo | M.P.H. | | Boston University School of Public Health | Boston, Massachusetts, US | Contributor |  |
| Joseph N. Palmisano | M.A., M.P.H. | | Boston University School of Public Health | Boston, Massachusetts, US | Contributor |  |
| Brittany Pine | B.A., B.S. | | Boston University School of Public Health | Boston, Massachusetts, US | Contributor |  |
| Janani Ramachandran | M.S. | | Boston University School of Public Health | Boston, Massachusetts, US | Contributor |  |
| Jennifer Fitzsimmons | M.D. | | Brigham and Women's Hospital | Boston, Massachusetts, US | Contributor |  |
| Elena M. Bonke | Ph.D. | | Brigham and Women's Hospital | Boston, Massachusetts, US | Contributor |  |
| Katherine Breedlove | Ph.D. | | Brigham and Women's Hospital | Boston, Massachusetts, US | Contributor |  |
| Eduardo Coello | Ph.D. | | Brigham and Women's Hospital | Boston, Massachusetts, US | Contributor |  |
| Hujiun Liao | B.S. | | Brigham and Women's Hospital | Boston, Massachusetts, US | Contributor |  |
| Elizabeth Rizzoni | B.A. | | Brigham and Women's Hospital | Boston, Massachusetts, US | Contributor |  |
| Vivian Schultz | M.D. | | Brigham and Women's Hospital | Boston, Massachusetts, US | Contributor |  |
| Annelise Silva | B.S. | | Brigham and Women's Hospital | Boston, Massachusetts, US | Contributor |  |
| Brynn Vessey | B.S. | | Brigham and Women's Hospital | Boston, Massachusetts, US | Contributor |  |
| Sarah Banks | Ph.D. | | Cleveland Clinic Lou Ruvo Center for Brain Health | Las Vegas, Nevada, US | Contributor |  |
| Jason Miller | Ph.D. | | Cleveland Clinic Lou Ruvo Center for Brain Health | Las Vegas, Nevada, US | Contributor |  |
| Aaron Ritter | M.D. | | Cleveland Clinic Lou Ruvo Center for Brain Health | Las Vegas, Nevada, US | Contributor |  |
| Marwan Sabbagh | M.D. | | Cleveland Clinic Lou Ruvo Center for Brain Health | Las Vegas, Nevada, US | Contributor |  |
| Raelynn de la Cruz |  | | Cleveland Clinic Lou Ruvo Center for Brain Health | Las Vegas, Nevada, US | Contributor |  |
| Jan Durant |  | | Cleveland Clinic Lou Ruvo Center for Brain Health | Las Vegas, Nevada, US | Contributor |  |
| Moragn Golceker |  | | Cleveland Clinic Lou Ruvo Center for Brain Health | Las Vegas, Nevada, US | Contributor |  |
| Nicolette Harmon |  | | Cleveland Clinic Lou Ruvo Center for Brain Health | Las Vegas, Nevada, US | Contributor |  |
| Kaeson Kaylegian |  | | Cleveland Clinic Lou Ruvo Center for Brain Health | Las Vegas, Nevada, US | Contributor |  |
| Rachelle Long |  | | Cleveland Clinic Lou Ruvo Center for Brain Health | Las Vegas, Nevada, US | Contributor |  |
| Priscilla Sandoval |  | | Cleveland Clinic Lou Ruvo Center for Brain Health | Las Vegas, Nevada, US | Contributor |  |
| Robert W. Turner III | Ph.D. | | George Washington University School of Medicine and Health Sciences | Washington, D.C., US | Contributor |  |
| Kenneth L. Marek | M.D. | | Invicro (formerly Molecular NeuroImaging) | Needham, Massachusetts, US | Contributor |  |
| Andrew Serrano | M.B.A. | | Invicro (formerly Molecular NeuroImaging) | Needham, Massachusetts, US | Contributor |  |
| David W. Dodick | M.D. | | Mayo Clinic Arizona | Phoenix, Arizona, US | Contributor |  |
| Yonas Geda | M.D., MSc | | Mayo Clinic Arizona | Phoenix, Arizona, US | Contributor |  |
| Jennifer V. Wethe | Ph.D. | | Mayo Clinic Arizona | Phoenix, Arizona, US | Contributor |  |
| Bryce Falk | R.N. | | Mayo Clinic Arizona | Phoenix, Arizona, US | Contributor |  |
| Amy Duffy |  | | Mayo Clinic Arizona | Phoenix, Arizona, US | Contributor |  |
| Marci Howard |  | | Mayo Clinic Arizona | Phoenix, Arizona, US | Contributor |  |
| Michelle Montague |  | | Mayo Clinic Arizona | Phoenix, Arizona, US | Contributor |  |
| Thomas Osgood |  | | Mayo Clinic Arizona | Phoenix, Arizona, US | Contributor |  |
| Debra Babcock | M.D., Ph.D. | | National Institute of Neurological Disorders and Stroke (NINDS) | Bethesda, Maryland, US | Administrative |  |
| William Barr | Ph.D. | | New York University | New York, New York, US | Contributor |  |
| Judith Goldberg | Sc.D. | | New York University | New York, New York, US | Contributor |  |
| Thomas Wisniewski | M.D. | | New York University | New York, New York, US | Contributor |  |
| Ivian Kirov | Ph.D. | | New York University | New York, New York, US | Contributor |  |
| Yvonne Lui | M.D. | | New York University | New York, New York, US | Contributor |  |
| Charles Marmar | M.D. | | New York University | New York, New York, US | Contributor |  |
| Lisene Hasanaj |  | | New York University | New York, New York, US | Contributor |  |
| Liliana Serrano |  | | New York University | New York, New York, US | Contributor |  |
| Alhassan Al-Kharafi |  | | New York University | New York, New York, US | Contributor |  |
| Sammie Martin |  | | New York University | New York, New York, US | Contributor |  |
| Edward Riley |  | | New York University | New York, New York, US | Contributor |  |
| William Runge |  | | New York University | New York, New York, US | Contributor |  |
| Elaine R. Peskind | M.D. | | University of Washington and VA Puget Sound | Seattle, Washington, US | Contributor |  |
| Elizabeth Colasurdo |  | | University of Washington and VA Puget Sound | Seattle, Washington, US | Contributor |  |
| Daniel S. Marcus | Ph.D. | | Washington University (CNDA) | St. Louis, Missouri, US | Contributor |  |
| Jenny Gurney | M.S. | | Washington University (CNDA) | St. Louis, Missouri, US | Contributor |  |
| Keith A. Johnson | M.D. | | Massachusetts General Hospital | Boston, Massachusetts, US | Contributor |  |
| Richard Greenwald | Ph.D. | | Simbex | Lebanon, New Hampshire, US | Contributor |  |
